# Novel Modeling of Combinatorial miRNA Targeting Identifies SNP with Potential Role in Bone Density

**DOI:** 10.1371/journal.pcbi.1002830

**Published:** 2012-12-20

**Authors:** Claudia Coronnello, Ryan Hartmaier, Arshi Arora, Luai Huleihel, Kusum V. Pandit, Abha S. Bais, Michael Butterworth, Naftali Kaminski, Gary D. Stormo, Steffi Oesterreich, Panayiotis V. Benos

**Affiliations:** 1 Department of Computational and Systems Biology, University of Pittsburgh, Pittsburgh, Pennsylvania, United States of America; 2 Fondazione Ri.MED, Palermo, Italy; 3 Department of Pharmacology and Chemical Biology, Womens Cancer Research Center, University of Pittsburgh School of Medicine, Pittsburgh, Pennsylvania, United States of America; 4 Dorothy P. and Richard P. Simmons Center for Interstitial Lung Disease, Division of Pulmonary, Allergy and Critical Care Medicine, University of Pittsburgh, School of Medicine, Pittsburgh, Pennsylvania, United States of America; 5 Department of Cell Biology and Physiology, University of Pittsburgh, Pittsburgh, Pennsylvania, United States of America; 6 Department of Genetics, Washington University School of Medicine, St. Louis, Missouri, United States of America; 7 Clinical and Translational Science Institute, University of Pittsburgh, Pittsburgh, Pennsylvania, United States of America; Rutgers University, United States of America

## Abstract

MicroRNAs (miRNAs) are post-transcriptional regulators that bind to their target mRNAs through base complementarity. Predicting miRNA targets is a challenging task and various studies showed that existing algorithms suffer from high number of false predictions and low to moderate overlap in their predictions. Until recently, very few algorithms considered the dynamic nature of the interactions, including the effect of less specific interactions, the miRNA expression level, and the effect of combinatorial miRNA binding. Addressing these issues can result in a more accurate miRNA:mRNA modeling with many applications, including efficient miRNA-related SNP evaluation. We present a novel thermodynamic model based on the Fermi-Dirac equation that incorporates miRNA expression in the prediction of target occupancy and we show that it improves the performance of two popular single miRNA target finders. Modeling combinatorial miRNA targeting is a natural extension of this model. Two other algorithms show improved prediction efficiency when combinatorial binding models were considered. ComiR (Combinatorial miRNA targeting), a novel algorithm we developed, incorporates the improved predictions of the four target finders into a single probabilistic score using ensemble learning. Combining target scores of multiple miRNAs using ComiR improves predictions over the naïve method for target combination. ComiR scoring scheme can be used for identification of SNPs affecting miRNA binding. As proof of principle, ComiR identified rs17737058 as disruptive to the miR-488-5p:NCOA1 interaction, which we confirmed *in vitro*. We also found rs17737058 to be significantly associated with decreased bone mineral density (BMD) in two independent cohorts indicating that the miR-488-5p/NCOA1 regulatory axis is likely critical in maintaining BMD in women. With increasing availability of comprehensive high-throughput datasets from patients ComiR is expected to become an essential tool for miRNA-related studies.

## Author Summary

MicroRNA genes (miRNAs) are small non-coding RNAs that regulate the expression levels of mRNAs post-transcriptionally. miRNAs are critical in many important biological processes, like development, and are important markers for many diseases. Identifying the targets of miRNAs is not an easy task. Recent developments of high-throughput data collection methods for identification of all miRNA targets in a cell are promising, but they still depend on computational algorithms to identify the exact miRNA:mRNA interactions. In this paper we present a novel algorithm, ComiR, which addresses a more general question, that is, whether a given mRNA is targeted by *a set* of miRNAs. ComiR uses miRNA expression to improve the targeting models of four target prediction algorithms. Then it combines their predicted targets using a support vector machine. By applying ComiR to single nucleotide polymorphism (SNP) data, we identified a SNP that is likely to be causally associated to osteoporosis in women.

## Introduction

MicroRNAs (miRNAs) belong to a class of short (18–25 nucleotide) non-coding RNAs that regulate gene expression post-transcriptionally. Their regulatory activity depends heavily on the recognition of target sites located primarily on the 3′-untranslated regions (3′UTRs) of messenger RNAs [1] but also on ORFs or 5′-UTRs [2]. In general, a gene contains multiple miRNA binding sites. Computational miRNA target prediction depends on algorithms that typically use features like Watson–Crick base pair matching [3]–[5], thermostability of binding sites [3], [6]–[12], accessibility of target sites [3], [13], and phylogenetic conservation [3], [5]. Still, target prediction algorithms suffer from high number of false predictions and poor overlap in their predictions [14], [15]. It is worth noting that most existing algorithms only utilize site-specific features [16] ignoring factors like the relative expression of miRNAs that affects binding specificity and target combinatorial effects. The level of miRNA expression affects which targets will be occupied. When a miRNA is expressed at low levels it is expected to bind to only few, high affinity targets. As miRNA expression is increased, and all high affinity targets are occupied, the remaining miRNA molecules can bind to suboptimal targets of moderate affinity (due to target exclusivity). We believe that the 1∶1 stoichiometric binding model used by existing algorithms may be insufficient for the dynamic nature of real miRNA:mRNA interactions. This may be a major drawback of the current target prediction algorithms. Another drawback of most algorithms is that they only consider single miRNA:mRNA pairings, ignoring the fact that multiple miRNAs, each with a moderate effect, can collectively alter significantly the expression levels of a given mRNA. Extending single pairing predictions to the union or intersection of targets of multiple miRNAs does not solve this problem since these models do not consider multiple moderate binding effects and they also tend to be either very conservative or having large numbers of false positive predictions, respectively. Until recently, the only notable exceptions were PicTar [10] and GenMir++ [17]. PicTar uses a hidden Markov model to determine targets of multiple miRNAs, but it does not consider the miRNA expression in determining the relative binding. GenMir++ implements an elaborate Bayesian framework to model miRNA:mRNA dependencies using expression data and prior targeting information. The prior information is provided in binary form (putative targets/non-targets) and the effect of multiple miRNAs on a given mRNA is inferred using an expectation maximization (EM) algorithm. TaLasso [18], a recently published algorithm, combines miRNA expression with prior database information to infer regulation of mRNAs from a set of miRNAs using a lasso regression method to determine the activities of various miRNAs. Finally, Jayaswal *et al.* [19] proposed another method for finding multiple (many-to-many) miRNA:mRNA interactions by following a two-step approach. First, they identify miRNA and mRNA expression clusters and in the second step they find associations between them. In this case, there is no quantitative modeling of particular interactions. These algorithms have in common that they use miRNA expression to drive the target prediction for sets of miRNAs, but none of them has an underlying suitable thermodynamic model to account for target exclusivity. Furthermore, none of these algorithms has been used before to quantitatively rank single nucleotide polymorphisms (SNPs) affecting miRNA targeting. In general, so far, the evaluation of miRNA-related SNPs in disease has been done with more straightforward approaches [20], [21].

Until recently, a major obstacle for developing comprehensive miRNA binding models was the small number of available experimentally validated target pairs. This is now changing with the development of new experimental approaches that promise to generate validated miRNA:mRNA target data in a high-throughput fashion. One such technique is based on immunoprecipitation (IP) of miRISC proteins (RNA-induced silencing complex) [22], which probes the abundance of target mRNA bound to a mature miRNA. Although the actual miRNA:target pairs are not determined by this technique, the analysis of such datasets combined with the relative abundance of mRNAs and miRNAs [23] is critical for understanding the true cooperative interactions and model them in a quantitative way. Crosslinking immunoprecipitation (CLIP) is another recently developed and promising method. Both HITS-CLIP [24] and PAR-CLIP [25] allow for the miRNA target region to be determined in a narrow window on the mRNA. The high-throughput datasets these methods provide are ideal for developing methods to help a better understanding of the miRNA:mRNA targeting process.

This paper addresses various issues related to miRNA targeting. First, we show that miRNA targets generally act additively in regulating mRNA expression. This was previously postulated but –to our knowledge– never put in test. Second, we show that using miRNA expression to appropriately weigh miRNA targets can result in efficient additive combinatorial models. To that extent we develop a novel thermodynamic model for miRNA binding, based on the Fermi-Dirac equation and we show that using this model improves prediction accuracy and target overlap of PITA [26] and miRanda [27]. Prediction efficiency of TargetScan [28] and mirSVR [29] is also improved by weighting multiple miRNA targets by miRNA expression and combining their target scores additively. Third, we use a Support Vector Machine (SVM) to combine the improved predictions of these four algorithms in ComiR (Combinatorial miRNA targeting), our novel algorithm that is designed to address the question of how likely is for a set of miRNAs with known expression levels to influence the expression of a given mRNA. The algorithm is tested on previously published miRISC protein IP independent datasets, ranging over three species, *D. melanogaster*, *C. elegans* and *H. sapiens*. Finally, we show that ComiR scoring scheme is suitable for ranking SNPs affecting miRNA binding. As a proof of principle, we used ComiR scoring for ranking the genes of the estrogen receptor (ER) pathway and we predicted that SNP rs17737058 would disrupt a miR-488-5p binding site in NCOA1. We subsequently confirmed the interaction *in vitro* and we found the SNP to be associated with decreased bone mineral density in two independent datasets. These results illustrate the predictive power of ComiR and that rs17737058 should be further studied as a risk factor for osteoporosis. This is an important ComiR application, since identifying functional DNA sequence variants will be critical in the era where plentiful information will become readily available for many diseases.

## Results

### Evidence for additivity in miRNA targeting

First, we examined whether cooperativity is an important factor in miRNA targeting. Let-7d, miR-30b and scrambled miRNA were transfected into human fetal lung fibroblast and microarray analysis was performed. Log_2_ of fold change (FC) in transcript abundance was measured with respect to scrambled miRNA transfection after let-7d transfection (FC_l_), miR-30b transfection (FC_m_) or let-7d/miR-30b co-transfection (FC_lm_). Differentially expressed mRNAs were identified with SAM analysis (qval<0.05) in each of the three experiments. In particular, 1,413 genes were significantly down-regulated post let-7d transfection (746 of them were only down-regulated in the let-7d transfection), 1,819 post miR-30b transfection (966 of them were only down-regulated in the miR-30b transfection) and 1,039 after both miRNAs were transfected. The 132 out of the 1,039 down-regulated genes were identified only in the co-transfection experiment, indicating that these were targets on which miRNAs act co-operatively. We used stepwise regression to test the potential miRNA additive effect on the fold change of the target genes. Our model is:
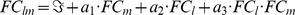
where ℑ is the intercept. We performed a backward elimination by eliminating the less significant variables. The elimination of the variable from the regression model always causes a decrease of the resulting R^2^ value of the regressions. We found that the let-7d and miR-30b mRNA interactions are additive for most genes: small decrease of the R^2^ value was observed when the cross product term was eliminated (from 67.1% to 67.05%; both p-val<10^−15^). However, when the regression model was applied to the subset of the 132 genes that were found to be significantly down-regulated only in the co-transfection experiment, then elimination of the cross product term significantly impacts the R^2^ value (p-value changes from 10^−3^ to 0.06). Notably, all weights obtained were <1, which probably reflects the saturation and competition effects on the miRISC machinery by the transfections [30]. The complete list of the regression coefficients is provided in Table S1.

Figure S1 presents the distribution of binding sites for the genes that were down-regulated in the single transfection experiments only (red and yellow bars), in all three experiments (green bars) and in the co-transfection experiment only (black bars). We see that in all but the last category a higher percentage of genes has few (1 or 2) binding sites, whereas for the genes were co-operativity was observed had generally more sites (4 or 7). This indicates that co-operativity may be more important when a large number of sites is present in a gene.

### Incorporating miRNA expression improves prediction efficiency of multiple targets

#### Considering miRNA expression in the thermodynamic binding model

**Figure 1:**
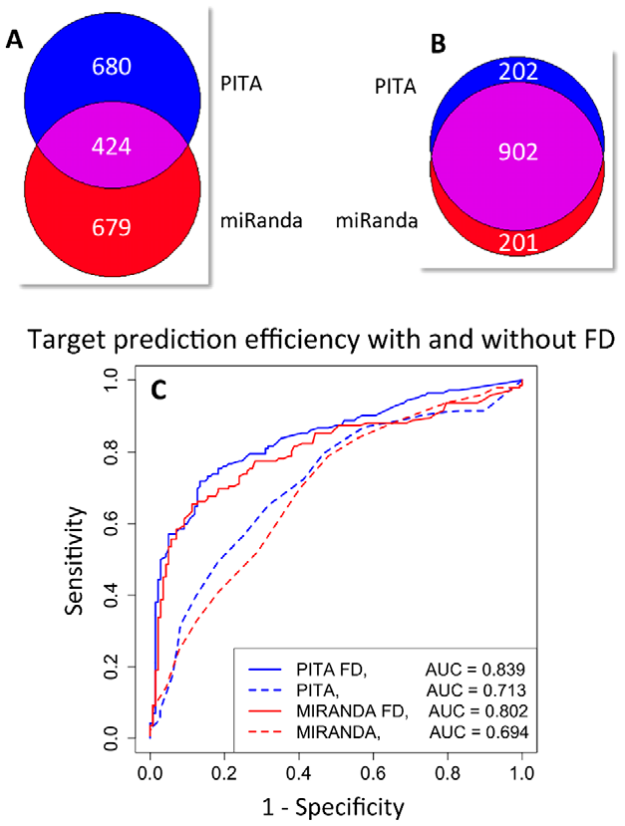
The effect of Fermi-Dirac model in miRNA target prediction. (A) Overlap of predicted targets from PITA and miRanda using a naïve combination of energy scores. (B) Target overlap between PITA and miRanda using the Fermi-Dirac energy score combination. (C) Receiver-operating Characteristic (ROC) curves of PITA and miRanda predictions with naïve (solid lines) and Fermi-Dirac (broken lines) energy score combination. AUC: area under the curve. Positive and negative sets were derived from the Ago1 IP data (Materials and Methods).

The thermodynamic model employed for miRNA:mRNA interactions in algorithms like PITA [26] and miRanda [27] assumes that binding occurs between a single miRNA and a single target in equilibrium. We developed a novel thermodynamic model based on the Fermi-Dirac (FD) equations, which takes into account both binding affinity and miRNA expression in determining the binding potential. The idea is similar to previous successful modeling of transcription factor binding activities performed by one of us [31] and others [32]. Suppose that miRNA *i* (*miR_i_*) has n_ik_ binding sites BS_ijk_ (j = 1,…,n_ik_) on mRNA *k*. The reversible reaction of binding of miR_i_ to the particular binding site BS_ijk_ is:
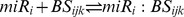



The equilibrium binding constant of the miR_i_ to the binding site BS_ijk_ is:
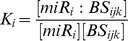
The probability of BS_ijk_ to be bound by miR_i_ is:
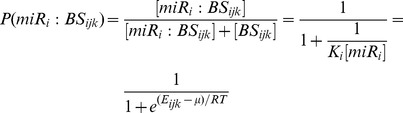
(1)where E_ijk_ = −RT• *ln*(K_i_) is the standard free energy of binding and *μ* = RT • *ln*([miR_i_]). The probabilistic score of Eq. 1 gives a natural way to combine the effect of multiple targets (from the same or multiple miRNA genes) on a given gene.
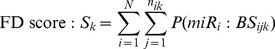
(2)For the gene *k* the FD score is the sum of probabilities over all binding sites (*n_ik_*) and all the considered miRNAs (*N*). In Eq. 1, as *E_ijk_* we can use the energy score provided by PITA or miRanda (typically negative values) and [miR_i_] is the concentration of miRNA *i*. As an estimate of the miRNA concentration in this paper we use its expression level. In this way, the binding sites with very negative energies and high expression level give the major contribution to the combined score. We call this method ***weighted score method*** to distinguish it from the ***naïve method***, according to which a gene is predicted to be a target of the miRNA set if it is a target of at least one of the miRNAs in the set. We compared the FD model to the naïve method on PITA and miRanda predictions of the targets of the 28 miRNAs in the Ago1-IP positive Drosophila dataset (see Materials and Methods). For both the naïve and the weighted score method, we considered the predicted genes associated with the top 10% of scores of each tool (i.e. ∼1100 genes) as targets. Although miRanda and PITA use related thermodynamic models, their overlap is poor (Figure 1A), reaching only 38% of the total predicted targets. When the FD model (Eq. 2) was used, the overlap was substantially improved, reaching 82% (Figure 1B). Using the positive and negative datasets (see Materials and Methods), we calculated the ROC curves of the performance of the two methods (Figure 1C) and we found that both sensitivity and specificity improves substantially with the use of the FD model for score combination. The AUC was improved by 18% and 16% in the case of PITA and miRanda, respectively.

#### Considering miRNA expression in other target prediction methods

Not all the target prediction algorithms use a thermodynamic model. TargetScan [28] for example uses a simple *n*-mer match and mirSVR [29] combines predictions of individual miRNA:mRNA targets from multiple algorithms using support vector regression. For these algorithms, miRNA expression can be used to weigh the score of target combinations (weighted sum score or WSUM) using Eq. 3:
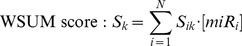
(3)where we sum the target scores S_ik_ detected for each miRNA, weighted by the relative expression level of the miRNA. Considering miRNA expression through Eq. 3 when combining miRNA targets improves the performance of TargetScan and mirSVR, although the improvement in this case is moderate (TargetScan AUC: 0.82 (WSUM) *vs.* 0.79 (naïve); mirSVR AUC: 0.82 (WSUM) *vs.* 0.80 (naïve)). In any case, these results show that regardless of the magnitude, incorporation of miRNA expression when combining targets of multiple miRNAs improves prediction efficiency of all four methods.

### ComiR: Integration of multiple prediction algorithms and miRNA expression data

**Figure 2:**
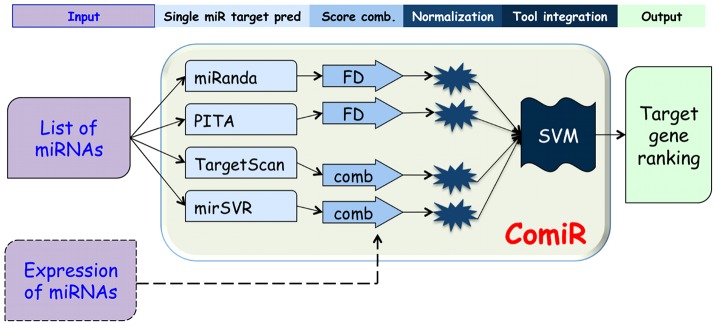
ComiR schema.

Based on the previous results, we developed ComiR (Combinatorial miRNA targeting), a novel algorithm that integrates the predictions of four top target prediction tools: PITA [26], miRanda [27], TargetScan [28] and mirSVR [29], into a single score. See Text S1 for more details about the implementation of the existing target prediction tools. First, each algorithm is run separately and for a given mRNA we identify all binding sites of each miRNA in its 3′UTR. Then, we incorporate miRNA expression and we additively combine the individual target scores using either Eq. 2 in the cases of PITA and miRanda (FD score), or Eq. 3 in the cases of TargetScan and mirSVR (WSUM score). By considering the miRNA expression in target score integration (Eq. 2 and Eq. 3) ComiR improves the efficiency of the corresponding algorithms as we showed above. The scores of the four tools for each mRNA are then combined through an SVM with linear kernel trained on the Drosophila RISC IP dataset [22] (see Materials and Methods). The target prediction score of ComiR is the class probability value computed by using the trained SVM model. Hence, ComiR scores range from 0 to 1 and higher scores correspond to higher probability of an mRNA being a functional target of the particular set of miRNAs. For the SVM implementation we used the ‘e1071’ R library. The general framework of ComiR is presented in Figure 2. The normalization step is a cross-species normalization of the score distributions (see Text S1 and Figure S2).

### ComiR outperforms existing algorithms within and across species

#### ComiR training and testing in Drosophila AGO1 IP datasets

**Figure 3:**
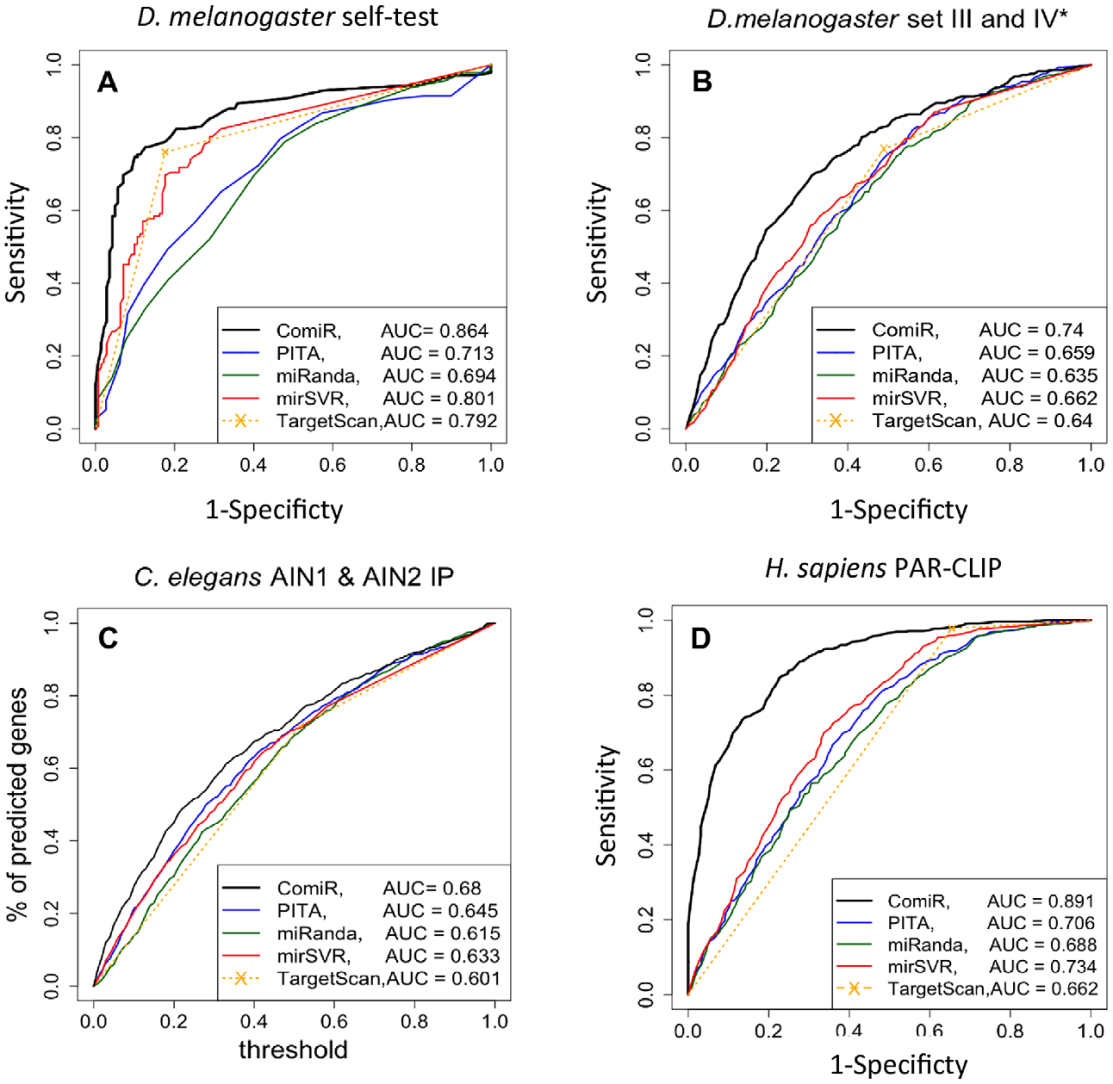
Predicting efficiency of Drosophila-trained ComiR on various datasets. (A) Self-test on the Drosophila Ago1-IP dataset consist of Set I (positive examples) and equal number of negative examples (from Set IV). (B) Performance on an external Drosophila Ago1-IP dataset consisting of Set III (positive examples) and the remaining of Set IV (negative examples). This Drosophila dataset was not used in training ComiR. (C) SN *vs.* threshold on an external *C. elegans* AIN-IP dataset (not an ROC curve due to inability to define a negative dataset). (D) Performance on an external human PAR-CLIP dataset. In all cases, TargetScan was used without the evolutionary conservation feature resulting in a binary outcome. For the human dataset the reader can find a continuous TargetScan ROC curve in Figure S3B, plotted using the *context score*.

ComiR was trained on the balanced Drosophila RISC IP dataset derived from [22], as we describe in Materials and Methods. In order to assess whether ComiR offers an improvement over the standard methods, we applied it to two Drosophila-derived datasets as well as independent datasets from *C. elegans* and humans. For the Drosophila, we first performed a self-test, which –as expected– showed ComiR to perform better than all other algorithms (Figure 3A; AUC = 0.85 compared to a low value of 0.69 for miRanda and a high value of 0.81 for mirSVR). Using the pROC package [33] we found that this difference was statistically significant for miRanda (p-value = 10^−5^) and PITA (p-value = 10^−3^). We also performed the leave-one-out-cross-validation (LOOCV) on this dataset with similar results (Figure S3A). Then we tested ComiR on a separate Drosophila dataset that was not used for training. This set consisted of Set III as positive examples and those Set IV genes that were not used for training as negative examples. Since none of these mRNAs was used in training, we consider this to be an external validation on a dataset from the same species. Interestingly, we found that all algorithms performed slightly worse, which might reflect to the less stringent conditions this set was derived from. Still, ComiR outperformed the other four algorithms with an AUC value that was 12–17% better (Figure 3B; AUC = 0.74 compared to a low value of 0.64 for miRanda and a high value of 0.66 for mirSVR). In the external Drosophila dataset, ComiR performance was statistically significantly higher compared to all four tools (p-values from 10^−8^ to 10^−16^).

#### Drosophila-trained ComiR predicting *C. elegans* targets

The *C. elegans* test data set includes a list of 49 miRNAs with known expression and two sets of mRNAs that are IP enriched with AIN1 and/or AIN2 proteins. We considered the 568 genes enriched in both AIN1 and AIN2 IP experiments as true targets (positive dataset), but in this case there is no suitable negative dataset. The Drosophila-trained ComiR was used to predict the targets of the 49 *C. elegans* miRNAs. Given a specific threshold, we compare the total number of genes predicted as functional targets in the whole set with the number of genes predicted as functional targets within the test set only. The enrichment of predicted genes in the test set is statistically significant, with a p-value lower than 10^−16^. We compared the results obtained with the naïve generalization of the other existing tools. Since we have no suitable negative examples in this dataset, we cannot calculate a proper ROC curve (nor a p-value). Thus, in Figure 3C we plot the percent of predicted targets versus the threshold and we calculate the AUC although we note that this is not a standard ROC curve. We observe that ComiR performs always better than the other four algorithms on the *C. elegans* IP enriched mRNA dataset. Out of the 49 miRNAs contained in this test set, only one (miR-79) was included in the Drosophila training set. Exclusion of this miRNA from the worm test set did not alter the results (data not shown).

#### Drosophila-trained ComiR predicting *H. sapiens* PAR-CLIP targets

PAR-CLIP [25] and HITS-CLIP [24] methods can provide useful datasets for testing the efficiency of our algorithm. In our analysis we considered the 27 most highly expressed miRNAs, which had been simultaneously blocked in the human PAR-CLIP experiment [25]. Furthermore, we assess the efficiency of target prediction tools by comparing the change in transcript's abundance, or fold change, with the target prediction tool's scores, under the hypothesis that functional targets of the 27 miRNAs are the ones with the highest change of decreasing transcript's abundance. None of these miRNAs was included in the Drosophila training dataset, thus the PAR-CLIP is another independent test set. First, we tested how the target prediction tools perform when the binding site search in the 3′UTRs is restricted on the Crosslink-Centered Regions (CCRs, see Material and Methods). Restricting the search region offers a considerable advantage to all prediction algorithms. ROC analysis showed that the Drosophila-trained ComiR model performs 22–35% better than the other target prediction tools in detecting human functional targets (Figure 3D; AUC = 0.89 compared to a low value of 0.66 for TargetScan and a high value of 0.73 for mirSVR). In the case of the human PAR-CLIP dataset, ComiR performed statistically significantly better compared to all four tools (p-values 10^−16^ for all algorithms). Unlike the other species where TargetScan returns a binary answer (without considering evolutionary conservation), for human data it provides a “context score”, which we used to plot a continuous ROC curve (Figure S3B). The plot showed that TargetScan context score provides a superior measure for collective target predictions over all other algorithms except ComiR. In fact, the AUC for ComiR was still 9.5% larger than that of TargetScan (p-value = 10^−12^). For completeness of the analysis, the precision-recall curves of the two Drosophila and the human datasets are presented in Figure S4. To perform the precision-recall curves we used the ‘ROCR’ R library [34].

Next, we considered the complete sequences of the 3′UTR, which is a broader but more commonly used dataset. In Figure S5, we report the empirical cumulative distribution function (ecdf) of the change in expression of the mRNAs after blocking the top 27 miRNAs. Genes containing at least one CCR in their 3′UTR sequence are grouped in deciles with respect to their target prediction score. In case of TargetScan, genes are grouped in two groups, predicted targets and predicted non-targets. We compare the ecdf of change in expression of mRNAs containing CCRs (colored lines) with the ecdf of mRNAs without CCRs (black line), by using the two-sample Kolmogorov-Smirnov (KS) distance and the Wilkoxon (W) rank test. The results are presented in Table S2. We observe that mRNAs with higher target prediction scores (1^st^ deciles) are always the farthest with respect to the reference distribution (Figure S5, black line and D values of the KS test (dKS) in Table S2), meaning that computational prediction tools are able to distinguish the functional targets. A comparison of the reference distribution of scores with the KS distances and the means of the ecdfs of the lower deciles shows that ComiR (normalized by rank) outperforms all the other considered tools (Table S2).

#### Drosophila-trained ComiR predicting miRNA transfection results in human lung fibroblasts

We run ComiR on the down-regulated genes resulting from the let-7d and miR-30b transfection and co-transfection experiments. Table S3 shows that for the top 20%, 25% and 30% of the predicted targets of each of the four tools, ComiR is generally more sensitive but less specific than each of them. mirSVR on the other hand is the most specific but the least sensitive in all cases. However, transfection experiments do not yield optimal datasets for testing miRNA target prediction, because of the severe perturbations they cause in the cell [30]. In such cases, miRNA-mediated gene regulation is not based solely on the miRNA:target binding energy, but also on the competition for the introduced and endogenous miRNAs for the available AGO proteins in the cell.

### Inclusion of miRNA expression drives the improvement in target prediction

**Figure 4:**
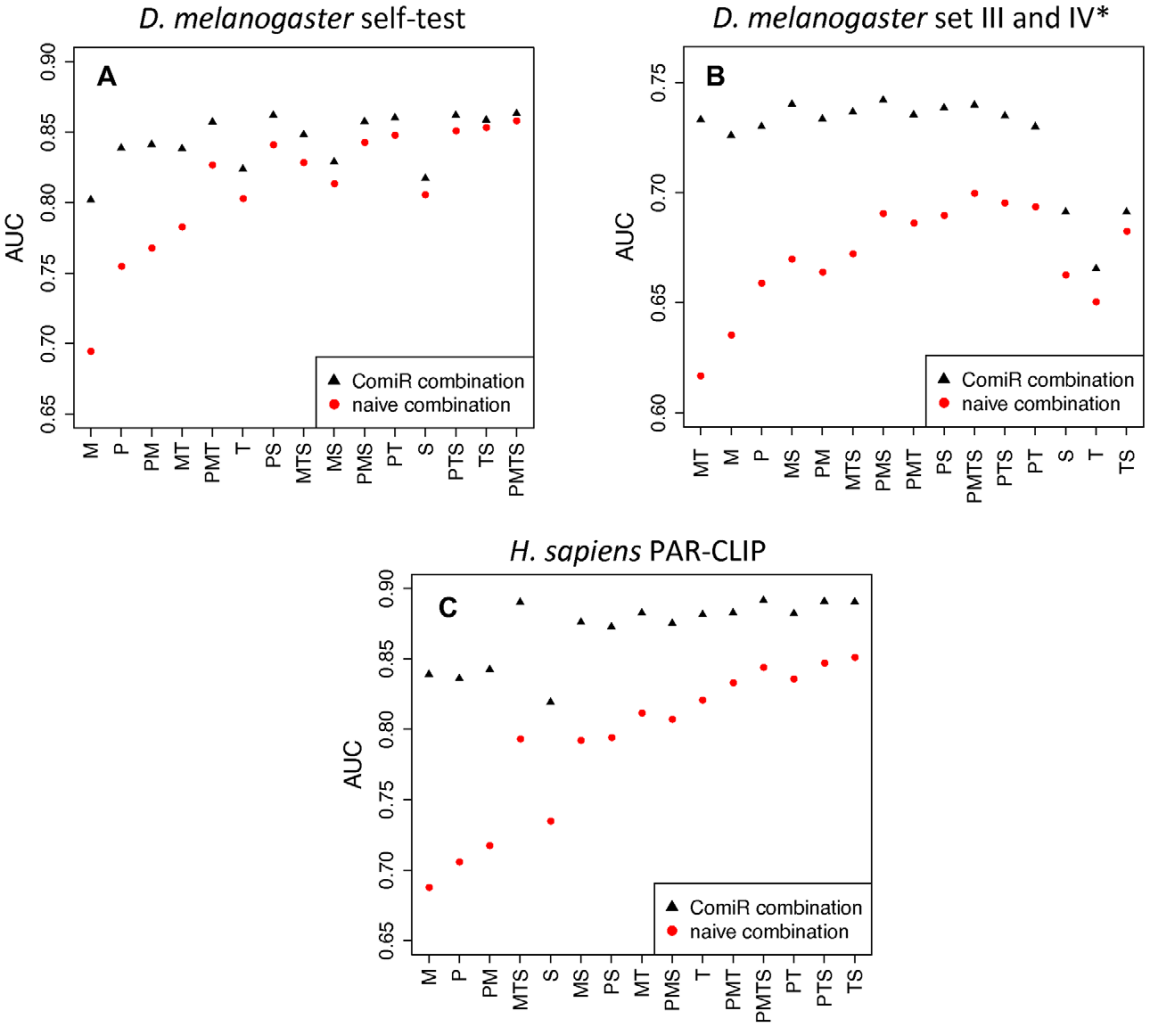
*Comparison of SVM models for multiple miRNA targets*. Multiple miRNA target scores are combined using the naïve model (red dots) or the ComiR model (FD score or WSUM score). The comparison has been performed on the same datasets as in Figure 3 with the exception of the *C. elegans* dataset, which has no proper ROC curve. Results are arranged by the difference the ComiR combination models offers over the naïve combination model. *P*: PITA, *M*: miRanda, *T*: TargetScan, *S*: mirSVR. AUC: area under the curve.

ComiR is a multi-step algorithm that calculates the probability of an mRNA being targeted by a set of miRNA genes. First, depending on the prediction tool, it uses the FD score (Eq. 2) or the WSUM score (Eq. 3) to incorporate the expression of each miRNA and to combine the scores of individual targets of the miRNA set in a single score. The FD score combination is used when the primary target finding tool is either miRanda or PITA (where binding energies are used for the scoring); whereas the WSUM score combination is used for TargetScan or mirSVR. Subsequently, an SVM is used to incorporate the prediction scores of the four individual tools into a single probabilistic score characteristic of the probability that this set of miRNAs target a particular mRNA. We investigated how much the ComiR score combination of multiple targets contributes to the improved performance over a naïve combination of scores. Figure 4 shows that considering miRNA expression through the FD score or WSUM score always improves the SVM integration of any combination of target finders, although the degree of improvement depends on the particular tool combination and the dataset used. In all three datasets the performance of the SVM with the improved combined scores of PITA, miRanda and TargetScan (PMT in Figure 4) is almost as good as the SVM with all four algorithms (Figure S6). The smallest improvement the ComiR score offers is for the TargetScan/mirSVR combination. Also, in the Drosophila external dataset, the ComiR score combination substantially improves the performance of most dual tool combinations (except TargetScan/mirSVR) and all the 3- and 4-tool combinations. In the human PAR-CLIP dataset we see that in general when scores are combined with the naïve model, TargetScan has the best performance, followed by mirSVR, PITA and miRanda. However, with the ComiR model for incorporation of miRNA expression (FD score or WSUM score) PITA and miRanda become better than mirSVR. Finally, in all datasets we see that the improvement of prediction accuracy is higher when the FD score is used (i.e., for of PITA and miRanda) than when the WSUM score is used (TargetScan and mirSVR). This indicates that the Fermi-Dirac model is indeed more accurate representation of the binding dynamics of miRNA:mRNA targeting, thus bringing the efficiency of PITA and miRanda closer to that of TargetScan and mirSVR. All the above indicate that incorporating miRNA expression in general and the Fermi-Dirac model in particular offer a very efficient way for combining individual target scores compared to the naïve model.

### ComiR predicts that rs17737058 disrupts an interaction critical for bone mineral density

**Figure 5:**
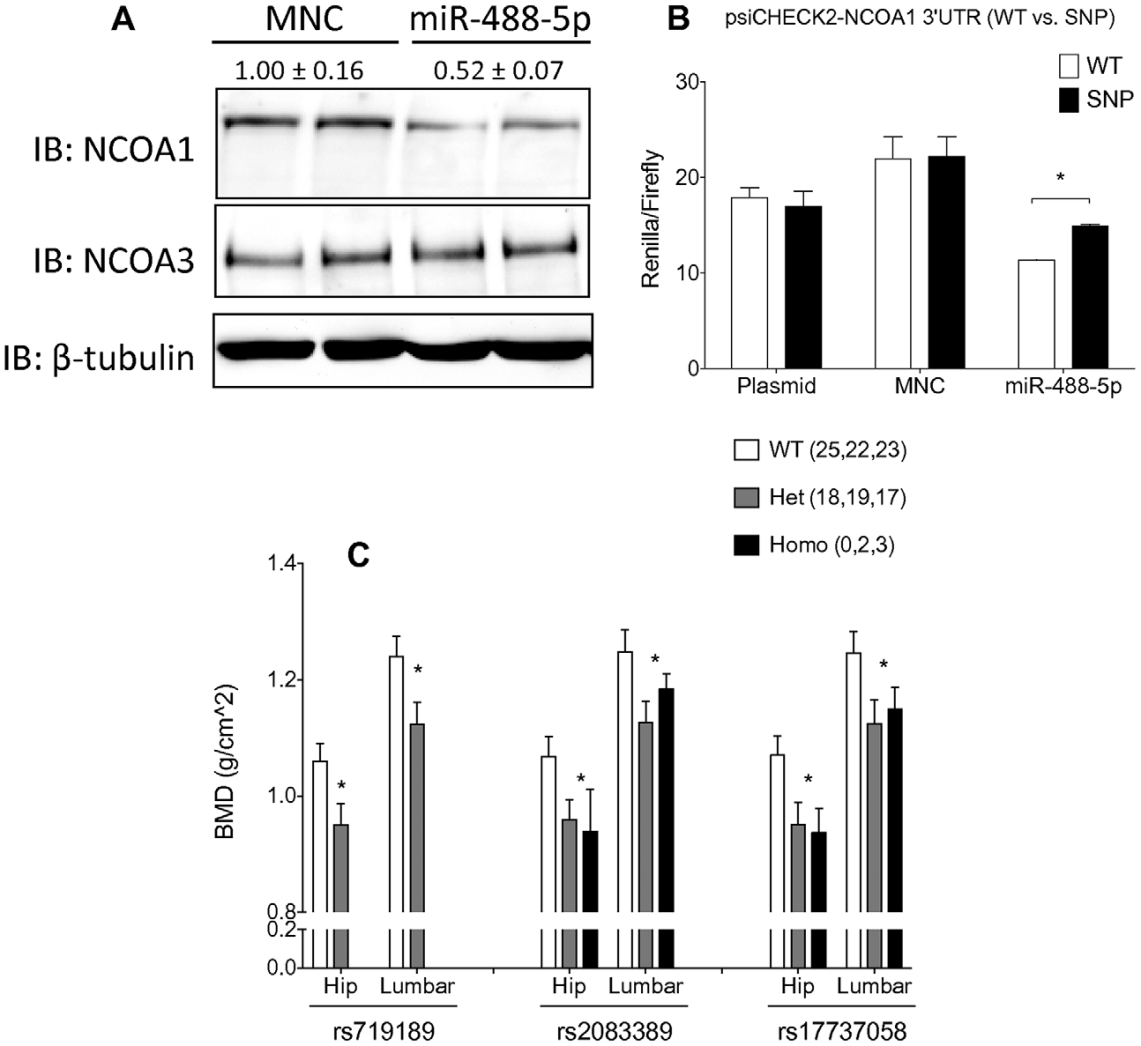
rs17737058 is sufficient to disrupt miR-488-5p targeting of NCOA1 (SRC-1) and it associates with BMD. (A) Western blot of U2OS-ERα cells were transfected with either a mimic negative control (MNC) or a miR-488-5p mimic miRNA. NCOA3 was used as a negative control and β-tubulin as a loading control. (B) U2OS-ERα cells were transfected with 100 nm of MNC or miR-488-5p mimic with 500 ng of either WT or SNP (rs17737058) psiCHECK2-renilla-NCOA1 3′UTR in combination with MNC or miR-488-5p miRNA renilla luciferase values were normalized to firefly luciferase values. * represents p<0.05 by t-test. (C) Premenopausal women not receiving bisphosphonates or chemotherapy (known role on BMD) in the COBRA trial were genotyped for association with decreased BMD. * p-values<0.05 comparing WT and Het+Homo BMD for each genotype by t-test.

The importance of the estrogen signaling, through the estrogen receptor α (ERα) pathway in bone maintenance is well established [35]–[38]. We asked the question whether ComiR could be applied to nine ERα pathway genes (Table S4) to identify SNPs in miRNA binding which could be associated with altered bone mineral density (BMD). Using dbSNP, we identified a total of 218 known SNPs in their 3′UTRs, 15 of which have minor allele frequency (MAF) greater than 10% (Table S4). There are about 400,000 SNP:miRNA pairs and nearly 29,000 of them correspond to the 15 SNPs with MAF>0.1. We ranked the SNP:miRNA pairs based on the SNP induced change in ComiR binding probability score. We wanted to focus on high confidence targets (those with high binding probability), so we used Eq. 4 for the ranking.
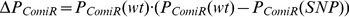
(4)Out of the ∼29,000 SNP:miRNA pairs we analyzed (those corresponding to the 15 SNPs of Table S4), 52 had 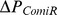
>0.01, with the miR-488-5p/rs17737058 (NCOA1) pair having the highest 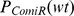 probability score among them. Importantly, rs17737058 is located in the center of the region matching the miR-488-5p seed sequence (Figure S7). We validated the effect of this SNP in the binding activity of miR-488-5p by first overexpressing a miR-488-5p mimic or mimic negative control (MNC) in U2OS-ERα cells and examining NCOA1 protein levels. Overexpression of miR-488-5p resulted in ∼50% relative reduction of NCOA1 levels (Figure 5A). As expected, no change was seen for NCOA3, a highly similar family member of NCOA1 that does not harbor a miR-488-5p target site (Figure 5A). Next, we examined if rs17737058 is sufficient to disrupt this regulation. Either the WT or rs17737058 3′UTR of NCOA1 was cloned downstream of the renilla luciferase CDS in the psiCHECK2 vector (Figure S8). The WT or SNP psiCHECK2 constructs were co-transfected with either an MNC or miR-488-5p. Consistent with the protein knockdown, WT renilla levels were reduced by ∼50% after overexpression of miR-488-5p (compared to MNC) (Figure 5B). However, SNP renilla showed a 30% attenuated miR-488-5p effect (Figure 5B). This indicates that the rs17737058 is sufficient to partially block the regulation of NCOA1 by miR-488-5p.

Since NCOA1 is known to modulate the estrogenic effect in bone, we further investigated the role of this SNP in osteoporosis by examining existing GWAS data. While rs17737058 is not present in most SNP-chips, three other SNPs in the same linkage-disequilibrium (LD) block (rs719189, rs2083389, and rs9309308) are represented on the Affymetrix 100k SNP-chip used in the Framingham Heart Study bone mineral density (BMD) genome-wide association study. All three SNPs are significantly associated with decreased BMD specifically in women (Table S5), but not at genome-wide significance levels so they were not included in the original publication [39]. To assess further the association between these SNPs and BMD, we genotyped for three of these NCOA1 SNPs in an independent patient cohort. We utilized germline DNA from a prospective clinical trial (COBRA) in which BMD was measured as part of a comprehensive phenotype characterization [40]–[42]. In support of the GWAS studies, we detected significant association between the three NCOA1 SNP and decreased BMD in premenopausal women (Figure 5C). In postmenopausal women (excluding patients taking bisphosphonates for treatment of osteoporosis as potential cofounding factor), we observed the same trend although it did not reach significance. Interestingly, examination of the postmenopausal women revealed that SNP carriers were more likely than expected to have been on bisphosphonates at the time of study (Table S6) suggesting that women with this SNP may have an increased risk of loss of BMD. A likely explanation of these data is that SNP carriers may experience premature bone loss before menopause that is less evident after menopause onset. This may be due to the underlying function of NCOA1 as a coactivator of ERα and therefore the phenotypes may be more pronounced in the presence of estrogen. Together these data indicate that rs17737058 can be associated with decreased BMD likely through the disruption of miR-488-5p regulation of NCOA1. However, further experimentation with animal models is required to prove this association. Thus, we showed that applying ComiR to the ER pathway resulted in the identification of a SNP in a miRNA:mRNA pair with clinical significance in hormone response in bone.

## Discussion

In this study we presented a new method that advances the miRNA target prediction field in two key areas. One, it considers the quantitative effect of miRNA expression in target occupancy using a new thermodynamic model; and two, it quantitatively evaluates and combines the effect of target sites of multiple miRNAs on a given mRNA. We showed that our methodology improves the efficiency of popular target prediction algorithms as well as the overlap of their target datasets. Combining these improved predictions in a single probabilistic score (*via* SVM methodology) resulted in a new algorithm, ComiR, which when trained on Drosophila AGO1-IP data, it efficiently predicted targets of the differentially expressed set of miRNAs in Drosophila, *C. elegans* and human.

By design, ComiR models the combinatorial effect of multiple miRNAs on a given mRNA. Thus, we expect that it will perform better in real-life examples, where multiple miRNAs are differentially expressed between two conditions. It is noteworthy that ComiR was proven to be more sensitive than any single tool. We attribute this to the nature of the SVM and the ComiR scoring system, which can elevate the targeting potential of a moderate affinity target predicted by any given tool if the miRNA is expressed in very high levels or if the mRNA contains multiple targets of this miRNA. Interestingly, TargetScan performed better than the other three algorithms and was competitive to ComiR on the single plotted point. However, ComiR remained the best of the algorithms tested. In addition, without evolutionary conservation TargetScan returns a binary outcome (target/no target) that does not allow for a threshold choice or for a scoring-based ranking of SNPs. The only exception is the human targets, where it provides a *context score*, which takes into consideration various features. Even in this case, ComiR was significantly more accurate in the human CLIP data and its better performance in terms of AUC was mostly in the region of high to medium false positive rate (Figure S3B). In any case, given the recent challenges of the assumption of evolutionary conservation of miRNA genes [43], [44] and their targets [45], methods that do not depend on evolutionary conservation may be proven a nice complement to the existing methods that do. Finally, in the high sensitivity area all algorithms seem to perform similarly, especially TargetScan and mirSVR. Notably, the SVM combination of TargetScan and mirSVR has the smallest improvement of ComiR *vs.* naïve combination of targets on this and the external Drosophila dataset (Figure 4).

Silencing of miRNAs is usually considered a milder perturbation in the cell than the one caused by transfection, because the transfected miRNAs that are introduced *en masse* in the cell create a challenge to the capacity of the miRNA loading machinery [30]. There are currently no data to facilitate modeling of the miRNA affinity to the mRISC complex. So, although the transfection experiments were not the ideal test bed for ComiR, it was still proven to be more sensitive than the other four algorithms.

We also showed that the ComiR score could be used to predict the effect of SNPs in single miRNA targets. Ideally, SNP and miRNA expression information should be obtained from the same individuals. We expect that such data will be routinely collected in the future. As a test case in this paper we analyzed the 3′UTR SNPs reported in dbSNP for the ERα pathway genes without having the benefit of knowing the miRNA expression levels. Based on ComiR top prediction, we postulated that miR-488-5p regulates NCOA1 and that rs17737058 reduces this regulatory effect. Since NCOA1 has a known role in maintaining BMD [35], [36], [38], we examined rs17737058 for an effect on BMD. Indeed, we found that this SNP was significantly associated with decreased BMD in two independent datasets. These results are strengthened by the observation that postmenopausal women carrying the SNP are more likely to be prescribed bisphosphonates than expected. This represents a rarely found example of a SNP disrupting a miRNA target site that results in a verified clinical phenotype. Interestingly, the clinical effect seems to be most evident in premenopausal women. We note that the FHS study was composed of two cohorts: the ‘Original Cohort’ (n = 159 women, mean age 77.5) and the ‘Offspring Cohort’ (n = 487 women, mean age 58.5) and the latter had three times more samples than the former. The other sub-studies within the osteoporosis GWAS meta-analysis [46] focused on older individuals, which might explain the failure to detect this association. Regardless, our data suggest that this SNP may identify premenopausal women at risk of osteoporosis and it should be considered a top candidate for further study and future development of personalized medicine therapeutic approaches. This is an important application of ComiR, because the identification of DNA sequence variants with a mechanistic functional role is becoming essential for the development of personalized medicine strategies.

Notably, PITA and miRanda ranked the SNP very far down the list (their score didn't practically change between wild-type and SNP sequence), mirSVR did not predict the pair, and TargetScan predicted the change but it offered no ranking. So, although in this case the lack of appropriate data did not allow us to take advantage of the full ComiR capabilities, it still provided a straightforward quantitative way to rank the SNPs affecting miRNA:mRNA interactions. We expect that in the future, when genotype and gene and miRNA expression data will be routinely collected from the same individuals, ComiR will be invaluable in identifying and ranking germline SNPs and somatic mutations that are associated to the disease.

In summary, ComiR, the novel miRNA target prediction method we presented here, solves two important problems that hinder miRNA target prediction and offers a quantitative way to rank SNPs associated to miRNA binding. To our knowledge, this is the first algorithm that models the detailed thermodynamic interactions of miRNA binding dynamics in a cell and incorporates the quantitative effect of miRNA expression on multiple targets of multiple miRNA genes on the same mRNA. ComiR is by no means perfect. miRNA targeting is a complicated procedure and many characteristics still remain unknown. mRNA cellular localization or association with various RNA-binding proteins may influence miRNA binding. Interplay between miR-328 and RNA binding proteins has been previously reported [47], but the data are still scarce to allow for efficient modeling. RNA folding can also play an important role in miRNA targeting as it appears to do in other biological phenomena [48]. New high-throughput datasets will become available in the future and help elucidate these interactions. In that respect, ComiR is the first step towards a more complete modeling of miRNA:mRNA interactions, which is expected to be improved further as more types of high-throughput data become available.

## Materials and Methods

### Training and test data sets

#### 3′UTR sequences

All the 3′UTR sequences used to implement our algorithm were selected from Ensembl.org. When the database contains more than one 3′UTR sequence for the same Ensembl ID, the longest sequence was selected. We considered only the 3′UTR sequences with at least 50 bases. In Table S7 we summarize the total number of genes and miRNAs considered for each analyzed species.

#### *Drosophila melanogaster* training and test data set

The primary data for the training set was obtained from the recent study [22]. Briefly, the authors performed an improved Ago1 IP protocol, identifying hundreds of miRNA targets in S2 cells of *Drosophila melanogaster* (fly). The resultant Ago1 IP data was compared with Ago1 depletion experiments [49] and less than 1/3 overlap was found between the mRNAs identified by the two experiments. Consequently, they divided mRNAs in the four sets schematized in Table S8. **Set I** is our positive training set consisting of 142 mRNAs who were bound to Ago1 and up-regulated following Ago1 depletion. **Set IV** contained the negative examples, consisting of the mRNAs that were not bound to Ago1, their expression remained unchanged following Ago 1 depletion, and have no predicted target sites [22], [50]. From Set IV we selected the 142 most highly expressed mRNAs to be our balanced negative training set. The identities and the expression values of the 28 miRNAs that had at least 50 reads in the S2 cells [23] were used in the ComiR input. The mRNA test set was composed of **Set III**, as positive examples, and the remaining genes in **Set IV** as negative examples. These datasets are not expected to be perfect positive or negative datasets. For example, Set III will contain mRNAs that were bound to Ago at low (undetectable) levels; but it will also contain secondary affected mRNAs (e.g., targets of TFs that were targeted by miRNAs). Also, the remaining Set IV genes are expressed at lower levels than those in the training set. For those reasons we expect this testing set to be noisier, but it is the best available we have to an external dataset.

#### *C. elegans* AIN IP test set

In Ref. [51], the authors use AIN-1 and AIN-2 IP in a mixed stage population of *C. elegans* followed by microarray analysis to identify potential miRNA targets. To test our approach on a *C. elegans* data set, we considered the list of 49 miRNA expressed with at least 50 reads in the cells [51] and the lists of mRNAs that were IP enriched in AIN-1 and AIN-2 IP. We use, as positive test set, the list of 568 3′UTRs of genes that were enriched in both AIN-1 and AIN-2 IP (set AIN1 and AIN2). Zhang *et al.* [51] only list the IP enriched mRNAs in AIN-1 and AIN-2 IP, so there is insufficient information to construct a negative test set with available data.

#### *H. sapiens* PAR-CLIP test set

Hafner *et al* [25] have published a PAR-CLIP (*P*hotoactivitable-*R*ibonucleoside-Enhanced *C*ross*l*inking and *I*mmuno*p*recipitation) dataset. RNA-binding proteins (RBPs) or ribonucleoprotein complexes (RNPs) were isolated in human embryonic kidney 293 cells (hek293). To facilitate crosslinking, transcripts of cultured cells were incorporated with 4-thiouridine (4SU) and RNA bound RBP binding sites were recorded by scoring for thymidine (T) to cytidine (C) transitions in the sequenced cDNA [25]. Region of about 41 nt, centered over these predominant T to C transitions were extracted. These Crosslink-Centered Regions (CCRs) constituted of clusters formed by at least 5 PAR-CLIP sequence reads and contained more than 20% T to C transitions [25]. To obtain evidence that CCRs contain functional miRNA binding sites, they blocked the 27 top expressed miRNAs in hek293 cells, and measured the change in mRNA expression.

We constructed our positive test set by considering all the 591 genes with at least one CCR located in the 3′ UTR of the gene and with a fold change after the 27 miRNA knockdown greater than 0.1. We only considered those genes for which 3′UTR sequence is available. We constructed the corresponding negative test set by choosing the same number of genes within the genes without CCRs lying in the 3′ UTR sequence, taking the ones with the highest average expression in untreated hek293 cells.

#### Human let-7d and/or miR-30b transfection

Human fetal lung fibroblasts (Lonza) were transfected with 100 nM let-7d precursor and/or miR-30b precursor (Ambion, Austin, TX). The results were compared to transfections with a negative control. 24 hours after transfection, RNA was isolated with the miRNeasy mini kit (Qiagen, Valencia). RNA quantity and quality were determined by NanoDrop and by Bioanalyzer (Agilent Technologies). Labeling was performed using the Agilent Low RNA Input Linear Amplification Kit PLUS, one color (5184–3523, Agilent Technologies) as per the manufacturer's instructions. Briefly, double-stranded cDNA was synthesized with an oligo(dT) primer, which later acts as a template for cRNA synthesis using T7 RNA polymerase. After verifying labeling efficiency, the Cy3 labeled cRNA was hybridized onto Agilent Whole Human Genome 4×44K arrays (G4112F, Agilent Technologies), with five replicates per each condition, washed and scanned using Agilent Microarray Scanner. Data files were obtained using Agilent Feature Extraction software version 9.5.3, data was cyclic lowess normalized and differentially expressed genes were identified using Significance Analysis of Microarrays (SAM) (http://www-stat.stanford.edu/~tibs/SAM). A q-value of 5 that corresponds to a false discovery rate (FDR) of 5% was set as the threshold for significance.

### ComiR application in identifying disease-related SNPs

#### SNPs in estrogen receptor (ER) pathway genes

SNPs in the 3′UTR sequence of NCOA1 were downloaded from the dbSNP database (human build 135) (Table S4). We considered the reference 3′UTR sequence (ref sequence) and 3′UTR containing each of the SNPs as separate sequences (SNP sequence). Each of these was analyzed with ComiR for potential binding differences for each of the known human miRNA genes (miRBase v. 18).

#### Assessing the effect of rs17737058 in miRNA targeting

To ensure that NCOA1 is indeed a target of miR-488-5p we overexpressed a miR-488-5p mimic to observe any changes in NCOA1 protein levels. The U2OS estrogen receptor-α stably transfected osteosarcoma cell line (U2OS-ERα: previously described, see [52]–[54]) were cultured in DMEM/F12 phenol red-free medium in 10% CSS and transfected with 50 nM of either a miRNA mimic negative control (MNC: CN-001000-01, Dharmacon) or a miR-488-5p mimic (488*: C-300748-05, Dharmacon) using Lipofectamine 2000 (Invitrogen) following the manufacturer's instructions. At ∼48 h following transfection, cells were harvested for western blot analysis. Antibodies used are as follows: anti-SRC-1 (128E7) (Cell Signaling: 2191S) and anti-α-tubulin (Sigma: T9026). To establish that rs17737058 is sufficient to disrupt miR-488-5p regulation of NCOA1 the wild-type 3′UTR of NCOA1 was cloned from MCF-7 cells to the multiple cloning site 3′ of *renilla* in the psiCHECK2 vector. Importantly, firefly luciferase is included in this vector under a separate promoter/polyA site and was used as a transfection control. Site directed mutagenesis was performed to generate the rs17737058 SNP construct. U2OS-ERα cells were transfected with 500 ng of either WT or SNP version of this construct with 100 nM of either a MNC or miR-488-5p mimic miRNA using Dharmafect Duo transfection reagent following the manufacturer's instructions. Cells were lysed ∼36 hours post-transfection and renilla/firefly luciferase activity was measured using the Dual Luciferase Assay System (Promega). Renilla activity was normalized to firefly luciferase.

#### Association of rs17737058 with decreased BMD

Since rs17737058 is not represented on most SNP-chips, we identified its linkage-disequilibrium (LD) block using HapMap-CEU data in the Genome Variation Server (GVS). This revealed a block of 28 SNPs in high LD with rs17737058. Three of these SNPs (rs2083389, rs719189, and rs9309308) are represented on the Affymetrix 100k SNP-chip used in the Framingham Heart Study (FHS) bone mineral density (BMD) study [39] downloaded from The Database of Genotype and Phenoptypes (dbGaP). Within this dataset, all SNPs specifically within NCOA1 (n = 7) were examined for an association with decreased BMD. Three of these SNPs were then genotyped with predesigned Taqman assays (Applied Biosystems) using germline DNA from a prospective tamoxifen breast cancer clinical trial, through the Consortium on Breast Cancer Pharacogenomics (COBRA) previously described (www.ClinicalTrials.gov; NCT0022893) [40]. This trial was designed to measure the effects of tamoxifen on various surrogates of estrogen activity. Only the baseline BMD tests performed before the administration of tamoxifen were used for the verification analysis. BMD was measured by standard dual-energy X-ray absorptiometry (DXA) scans, and the data have been deposited on http://www.pharmgkb.org with the accession ID PS207749 [55].

### Data access

The microarray data described in this work are deposited in the Gene Expression Omnibus database (GEO acc no.: GSE38530). A public web server for ComiR is available from the laboratory's web page: http://www.benoslab.pitt.edu/comir/.

## Supporting Information

Figure S1 **Distributions of the number of binding sites identified on downregulated genes in the transfection and co-transfection experiments.** Binding sites identified on genes downregulated only in the miR-30b transfection (red bars), only in the let-7d transfection (yellow bars), only in the co-transfection experiment (black bars), or in all three experiments (green bars).
(PDF)

Figure S2 **Species-specific score characteristics of four target prediction tools.** Density distribution of (A) the binding energy of all the binding sites as calculated with miRanda, (B) the interaction energy (ddG) os all the binding sites as calculated by PITA, (C) all the mirSVR scores of the conserved predicted target sites, and (D) the number of binding sites predicted by TargetScan. Red: *D. melanogaster*; green: *C. elegans*; blue: *H. sapiens*.
(JPG)

Figure S3 **Additional ROC curves.** **(A)** Leave-one-out-cross-validation (LOOCV) for the Drosophila AGO1 IP training dataset and **(B)** ROC curves include the *context score* of TargetScan for the human CLIP data.
(PDF)

Figure S4 **Precision-recall curves for the three datasets.** Results presented for the two Drosophila AGO1 IP datasets (self-test and external dataset) and the independent human PAR-CLIP dataset plotted in Figure 3. The *C. elegans* dataset was omitted since it did not have negative examples.
(PDF)

Figure S5 **Analysis of the PAR-CLIP CCR data.** ECDF of the change in expression after blocking the top 27 miRNAs of the mRNAs containing at least one CCR in the 3′ UTR sequence. Predictions are made by restricting the binding site searching on the CCR sequences. Lower deciles refer to higher probability to be a target. Genes are grouped in deciles respect to (A) ComiR with normalized scores, (B) ComiR with scores normalized by mean, (C) miRanda, and (D) PITA target prediction scores. In case of (E) mirSVR scores and (F) TargetScan scores, genes are divided in two groups, i.e. genes with and without seed's matching.
(PDF)

Figure S6 **Comparison of SVM models for multiple miRNA targets.** Multiple miRNA target scores are combined using the naïve model (red dots) or the ComiR model (FD score or COMB score). The comparison has been performed on the same datasets as in Fig. 3 with the exception of the *C. elegans* dataset, which has no proper AUC curve. The tool combinations are ordered by ComiR score combination performance. *P*: PITA, *M*: miRanda, *T*:TargetScan, *S*: mirSVR. AUC: area under the curve.
(PDF)

Figure S7 **Linkage disequilibrium in NCOA1 reveal potential function SNP driving BMD association.** Linkage disequilibrium (LD) r^2^ values were calculated using HapMap-CEU data using the Genome Variation Server (GVS). Arrows represent the two SNPs (rs719189, rs2083389) associated with decreased BMD in the FHS study. SNPs are arranged by LD bins represented by solid black horizontal lines. Both rs719189 and rs2083389 are found in the same LD bin with an additional 20 other SNPs at an average frequency of 21%. SNP rs number in green: synonymous, orange: 3′UTR. * represent SNPs where r^2^ values for all SNPs within a given bin are greater than 0.8.
(PDF)

Figure S8 ***Renilla luciferase*** **construct.** NCOA1 3′UTR was cloned into psiCHECK2 behind the renilla CDS.
(PDF)

Table S1 **Regression Coefficient resulting from a backward elimination of coefficient in the regression equation.** The “selected genes” set contains the genes that were down regulatedin the co-transfection experiment (miR-30b and let-7d) only, and not in the single transfection experiments. NC = not calculated (because eliminated from the model).
(XLSX)

Table S2 **PAR-CLIP** ***H.sapiens*** **ecdf analysis details.** For each considered target prediction tool (ComiR, miRanda, PITA, TargetScan and mirSVR) we report the mean, the standard deviation of the mean (sdm), the number of genes, the pvalue (pKS) and D value (dKS) of the KS test, and the W rank test pvalue (pW) of each decile (or group) of genes. Genes containing CCRs in the 3′UTR sequence are grouped by deciles with respect to their binding score for the considered target prediction tool. KS test and W rank test are performed by comparing each decile or group of genes with the reference set composed by genes that don't contain CCR in the 3′UTR sequence. Columns B–F contain results obtained by restricting the binding site search on CCR. Columns G–K contain the results obtained by analyzing the complete 3′UTR sequence.
(XLSX)

Table S3 **Comparison of ComiR to TargetScan and PITA on the let-7d/miR-30b transfection and co-transfection datasets.** Performance was measured on the set of down-regulated genes in each experiment.
(XLSX)

Table S4 **Information about SNPs examined as potentially altering miRNA targets.** ER pathway genes, number of SNPs in their 3′UTRs and common SNPs detected in their 3′UTRs. MAF: Minor Allele Frequency (as reported in dbSNP NCBI database)
(XLSX)

Table S5 **SNPs in NCOA1 associate with decreased BMD.** Due to the known role of NCOA1 in maintaining bone mineral density, data from the Framingham Heart Study (FHS) bone mineral density study was specifically examined for NCOA1 SNP associations with decreased BMD. Shown in the table are 2/6 SNPs within NCOA1 that show significant association with decreased BMD by either Familial Based Association Testing analysis (FBAT) or by Generalized Estimating Equations (GEE). P-values shown have been Bonferroni adjusted for 6 tests (based on 6 SNPs in NCOA1 represented in the SNP-chip used in this study). Other SNPs in NCOA1 not associated with decreased BMD and their MAFs are: rs2165739 (0.50), rs6724282 (0.05), rs6759706 (0.06), rs1992499 (0.4). MAF: minor allele frequency.
(XLSX)

Table S6 **Postmenopausal NCOA1 SNP carriers are more likely to be treated with bisphosphonates.** At time of study, more postmenopausal women with rs17737058 were found to be on bisphosphonates than expected by chance. No premenopausal women were on bisphosphonates at baseline. Although no significant BMD difference was seen between WT/SNP postmenopausal individuals, this is consistent with SNP carriers having increased clinical indications for bisphosphonates.
(XLSX)

Table S7 **Databases used and number of considered genes.**
(XLSX)

Table S8 **Sets of genes detected by comparing AGO1 IP and AGO1 depletion experiments.** We include the number of genes detected by Hong et al and the number of used 3′UTR sequences
(PDF)

Text S1 **Detailed description of ComiR implementation and other supporting information.**
(PDF)
