# Supplementary figures and images for: Novel Modeling of Combinatorial miRNA Targeting Identifies SNP with Potential Role in Bone Density
Source: PLoS Comput Biol. 2012 Dec 20;8(12):e1002830. doi: 10.1371/journal.pcbi.1002830 (PMC3527281; doi:10.1371/journal.pcbi.1002830)

# TargetScan

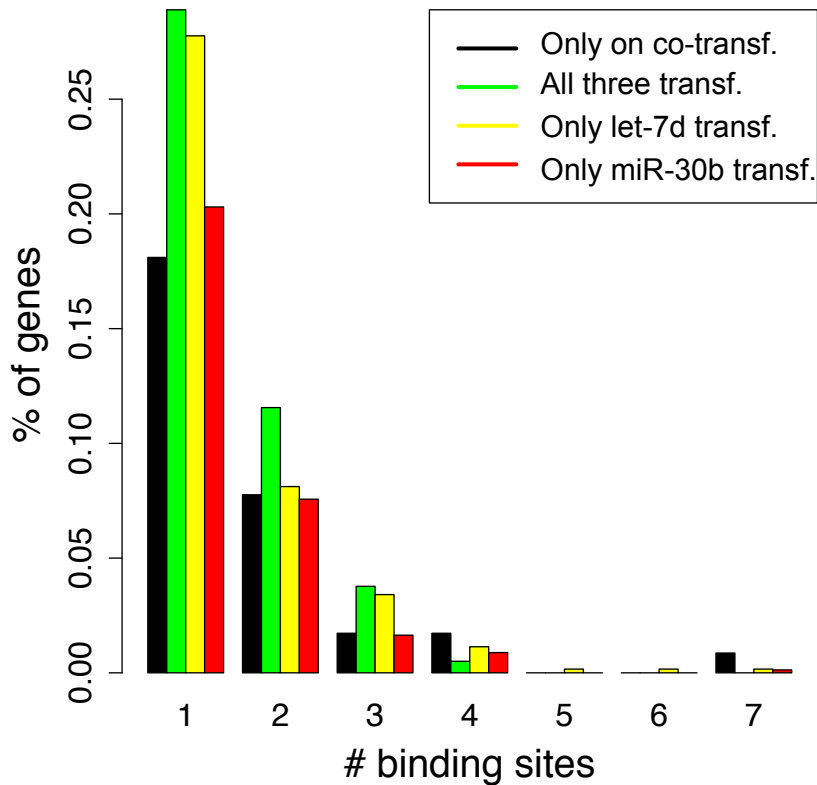

Supplement: Figure S1 — Distributions of the number of binding sites identified on downregulated genes in the transfection and co-transfection experiments. Binding sites identified on genes downregulated only in the miR-30b transfection (red bars), only in the let-7d transfection (yellow bars), only in the co-transfection experiment (black bars), or in all three experiments (green bars). (PDF) [file pcbi.1002830.s001.pdf]

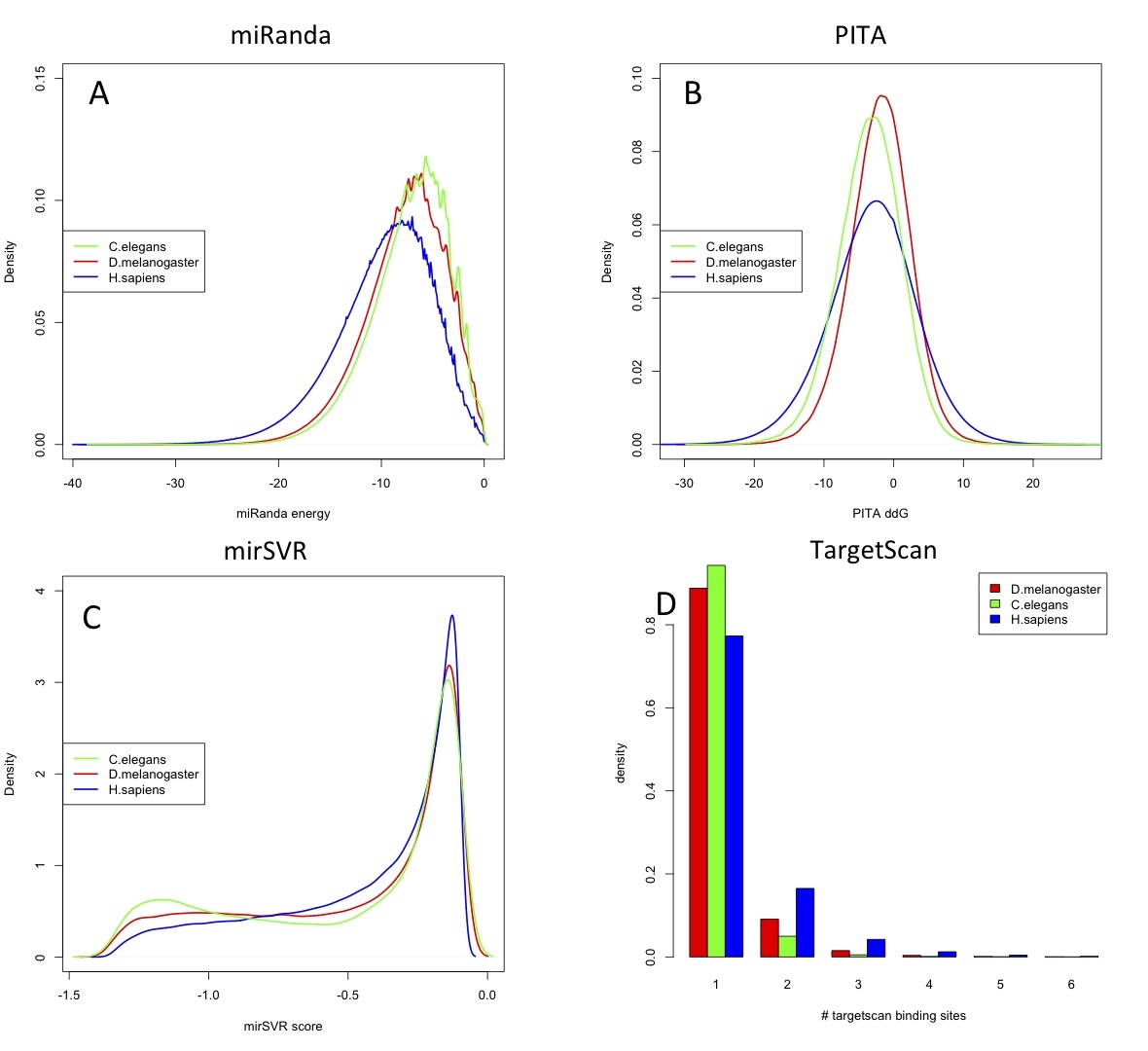

Supplement: Figure S2 — Species-specific score characteristics of four target prediction tools. Density distribution of (A) the binding energy of all the binding sites as calculated with miRanda, (B) the interaction energy (ddG) os all the binding sites as calculated by PITA, (C) all the mirSVR scores of the conserved predicted target sites, and (D) the number of binding sites predicted by TargetScan. Red: D. melanogaster; green: C. elegans; blue: H. sapiens. (JPG) [file pcbi.1002830.s002.jpg]

**A.****ComiR - LOOCV**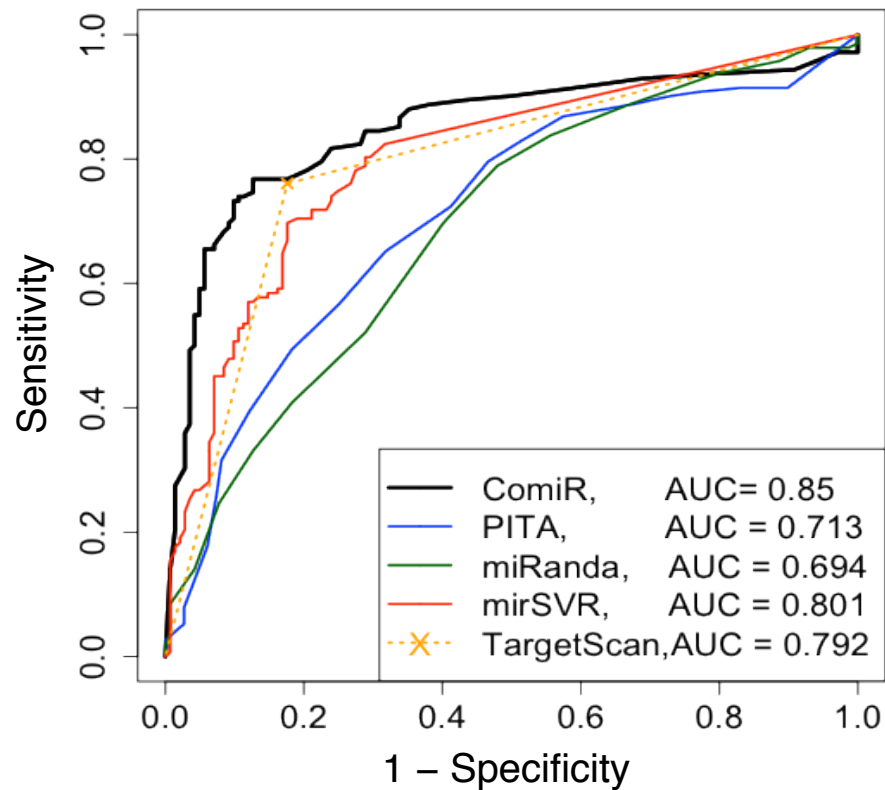**B.****ROC with TargetScan context score**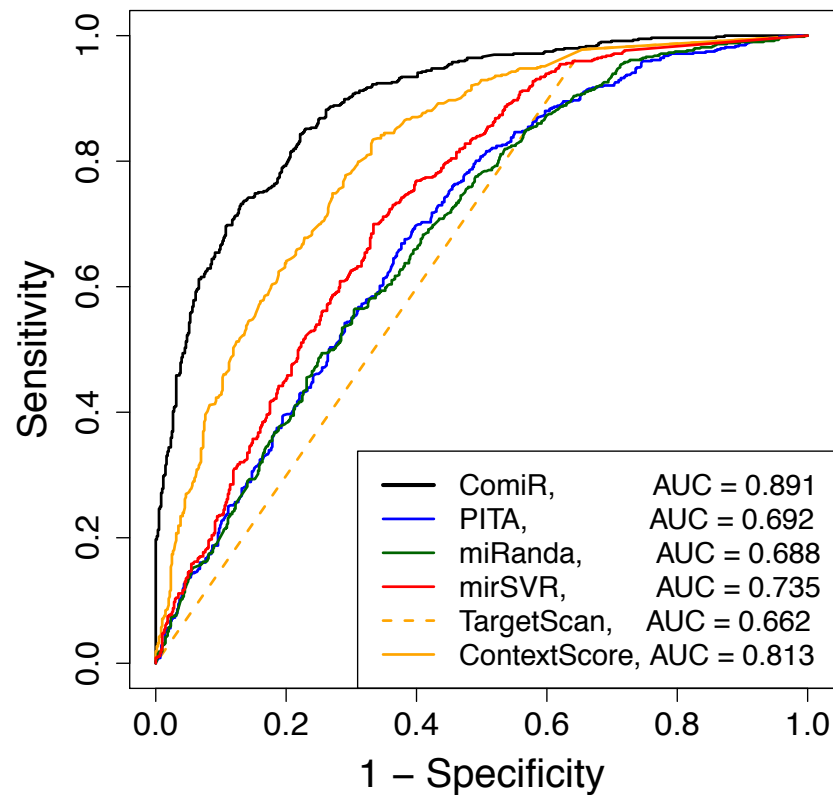

Supplement: Figure S3 — Additional ROC curves. (A) Leave-one-out-cross-validation (LOOCV) for the Drosophila AGO1 IP training dataset and (B) ROC curves include the context score of TargetScan for the human CLIP data. (PDF) [file pcbi.1002830.s003.pdf]

*D. melanogaster* self-test

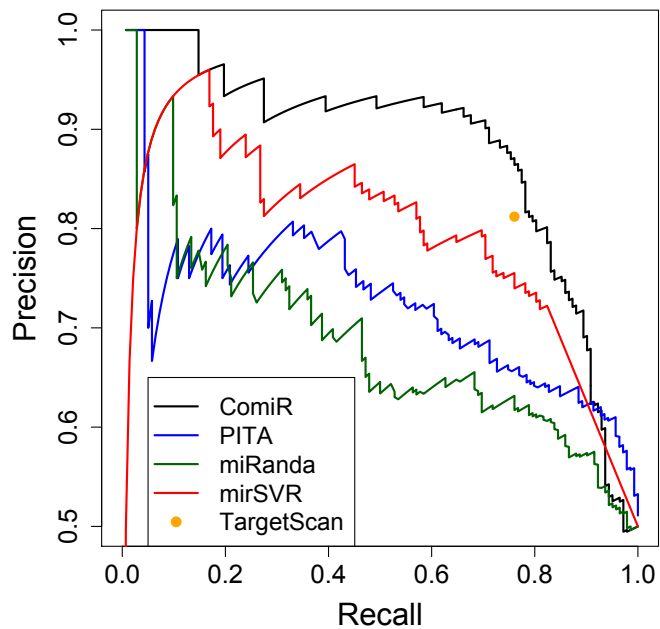

*D. melanogaster* set III and IV\*

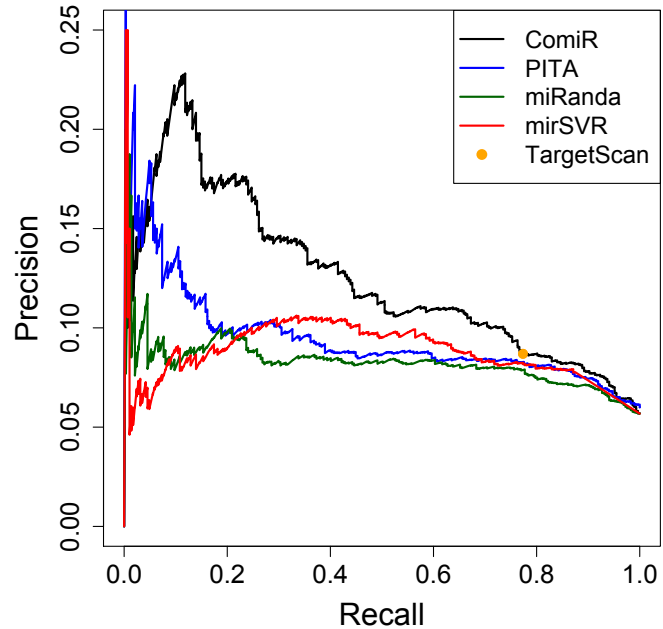

*H. sapiens* PAR-CLIP

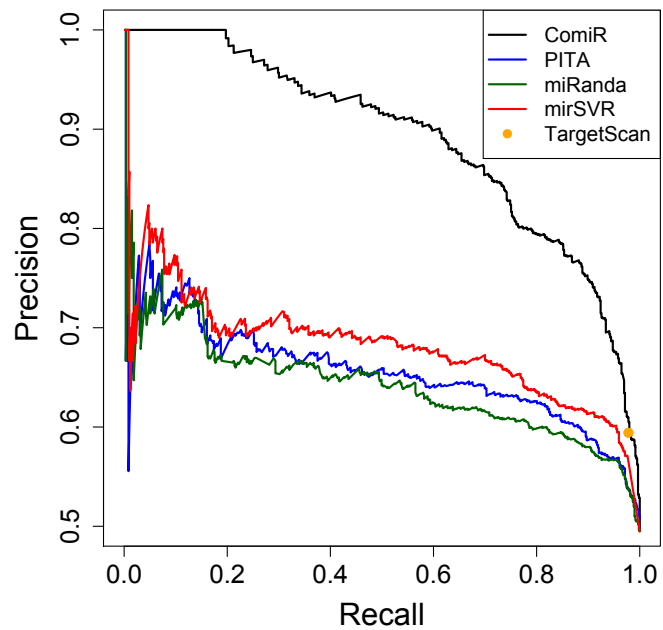

Supplement: Figure S4 — Precision-recall curves for the three datasets. Results presented for the two Drosophila AGO1 IP datasets (self-test and external dataset) and the independent human PAR-CLIP dataset plotted in Figure 3. The C. elegans dataset was omitted since it did not have negative examples. (PDF) [file pcbi.1002830.s004.pdf]

**ComiR**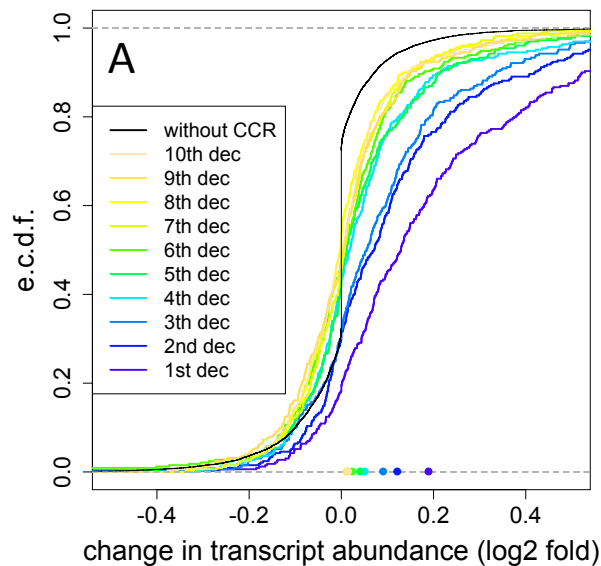**miRanda**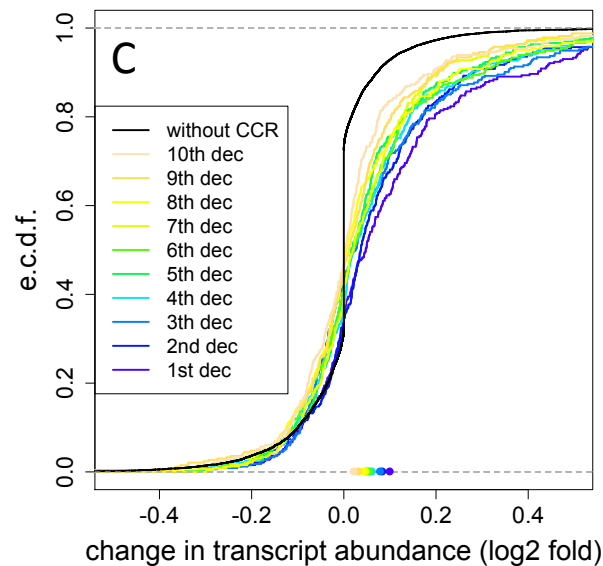**mirSVR**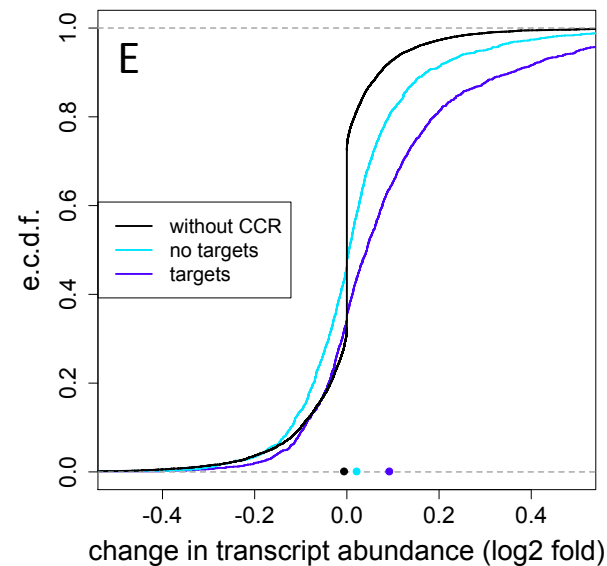**ComiR**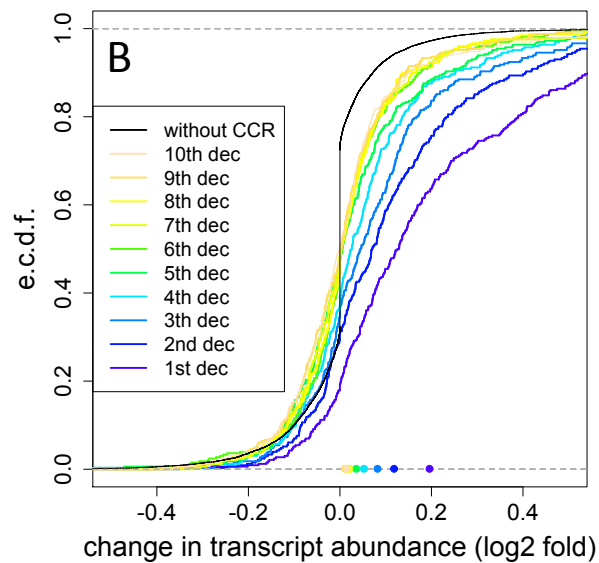**PITA**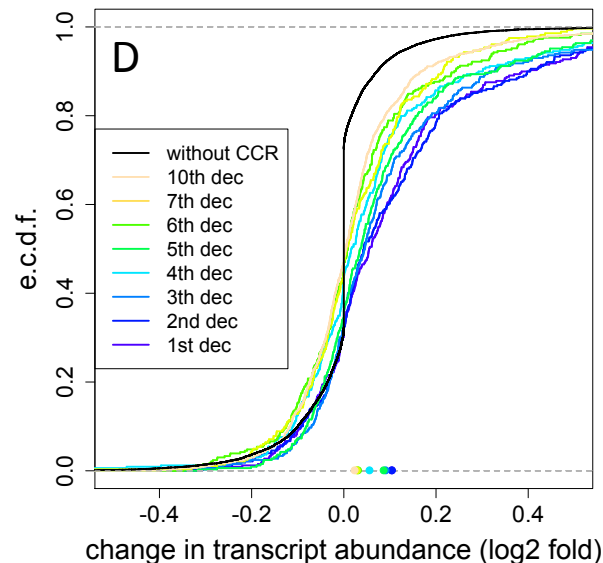**TargetScan**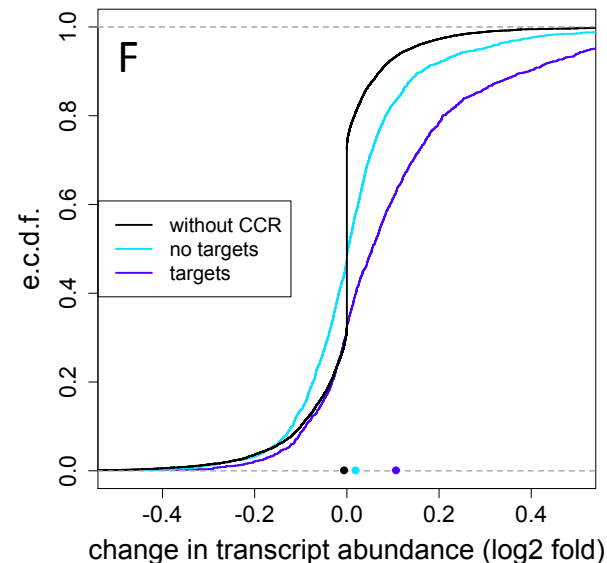

Supplement: Figure S5 — Analysis of the PAR-CLIP CCR data. ECDF of the change in expression after blocking the top 27 miRNAs of the mRNAs containing at least one CCR in the 3′ UTR sequence. Predictions are made by restricting the binding site searching on the CCR sequences. Lower deciles refer to higher probability to be a target. Genes are grouped in deciles respect to (A) ComiR with normalized scores, (B) ComiR with scores normalized by mean, (C) miRanda, and (D) PITA target prediction scores. In case of (E) mirSVR scores and (F) TargetScan scores, genes are divided in two groups, i.e. genes with and without seed's matching. (PDF) [file pcbi.1002830.s005.pdf]

*D. melanogaster* self-test

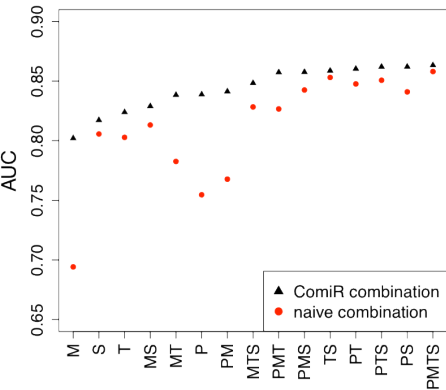

*D. melanogaster* set III and IV\*

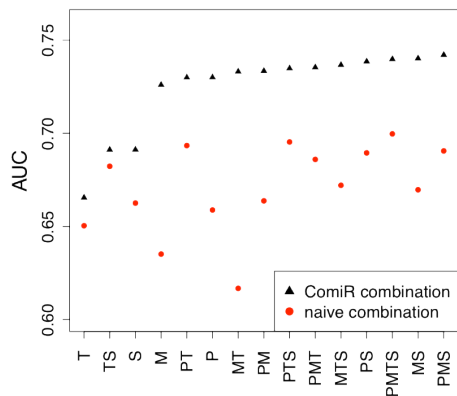

*H. sapiens* PAR-CLIP

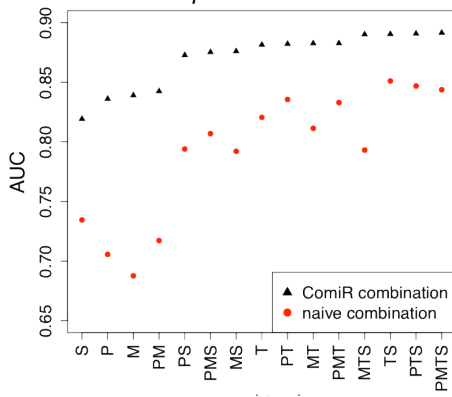

Supplement: Figure S6 — Comparison of SVM models for multiple miRNA targets. Multiple miRNA target scores are combined using the naïve model (red dots) or the ComiR model (FD score or COMB score). The comparison has been performed on the same datasets as in Fig. 3 with the exception of the C. elegans dataset, which has no proper AUC curve. The tool combinations are ordered by ComiR score combination performance. P: PITA, M: miRanda, T:TargetScan, S: mirSVR. AUC: area under the curve. (PDF) [file pcbi.1002830.s006.pdf]

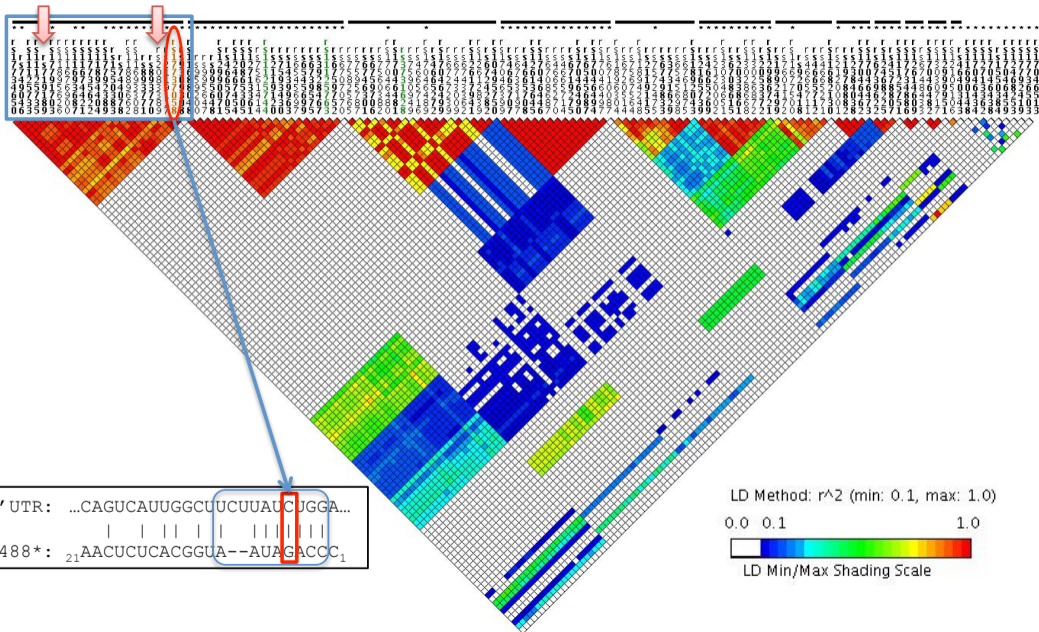

SRC-1 3' UTR: ...CAGUCAUUGGCUUCUUAUCUGGA...

miR488\*:  
 21 AACUCUCACGGUA--AUAGACCC<sub>1</sub>

Supplement: Figure S7 — Linkage disequilibrium in NCOA1 reveal potential function SNP driving BMD association. Linkage disequilibrium (LD) r2 values were calculated using HapMap-CEU data using the Genome Variation Server (GVS). Arrows represent the two SNPs (rs719189, rs2083389) associated with decreased BMD in the FHS study. SNPs are arranged by LD bins represented by solid black horizontal lines. Both rs719189 and rs2083389 are found in the same LD bin with an additional 20 other SNPs at an average frequency of 21%. SNP rs number in green: synonymous, orange: 3′UTR. * represent SNPs where r2 values for all SNPs within a given bin are greater than 0.8. (PDF) [file pcbi.1002830.s007.pdf]

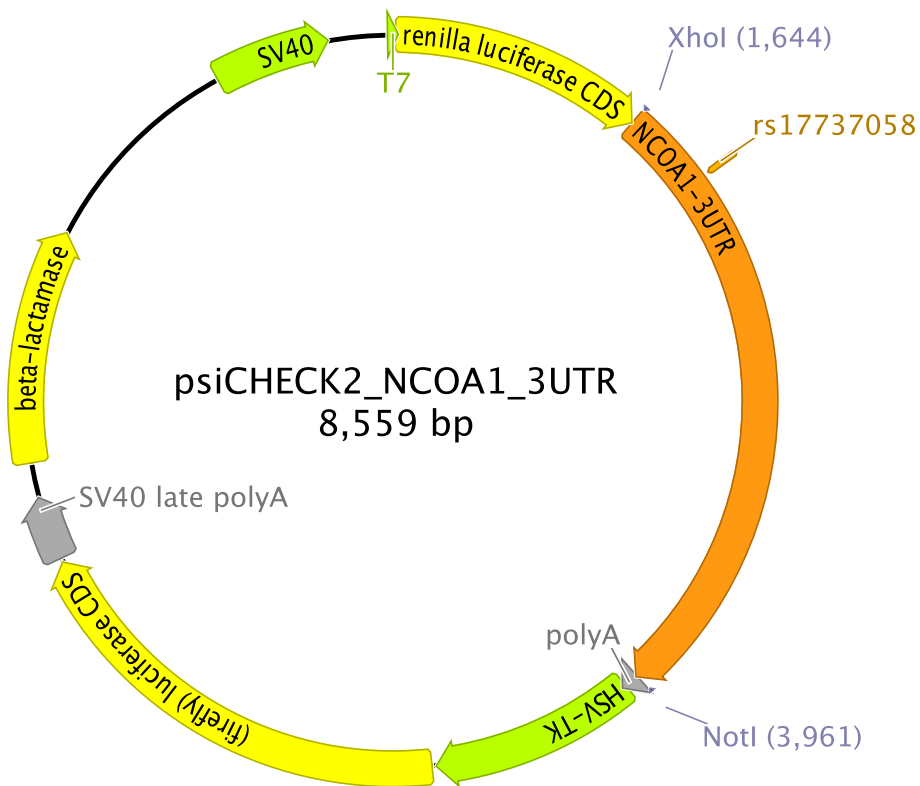

Supplement: Figure S8 — Renilla luciferase construct. NCOA1 3′UTR was cloned into psiCHECK2 behind the renilla CDS. (PDF) [file pcbi.1002830.s008.pdf]
