# Supplementary material for: Novel Modeling of Combinatorial miRNA Targeting Identifies SNP with Potential Role in Bone Density
Source: PLoS Comput Biol. 2012 Dec 20;8(12):e1002830. doi: 10.1371/journal.pcbi.1002830 (PMC3527281; doi:10.1371/journal.pcbi.1002830)
Supplement: Table S8 — Sets of genes detected by comparing AGO1 IP and AGO1 depletion experiments. We include the number of genes detected by Hong et al and the number of used 3′UTR sequences (PDF) [file pcbi.1002830.s016.pdf]

Supplementary Table S8. Sets of genes detected by comparing Ago1 IP and Ago1 depletion experiments. We include the number of genes detected by Hong et al and the number of used 3'UTR sequences

		AGO1 IP experiment	
	# genes / # 3'UTR	enriched	not enriched
AGO1 depl. experiment	up-regulated	**SET I** 152 / 142	**SET III** 300 / 287
	not up-regulated	**SET II** 1039 / 949	**SET IV** 5509 / 4907
