# Supplementary material for: Novel Modeling of Combinatorial miRNA Targeting Identifies SNP with Potential Role in Bone Density
Source: PLoS Comput Biol. 2012 Dec 20;8(12):e1002830. doi: 10.1371/journal.pcbi.1002830 (PMC3527281; doi:10.1371/journal.pcbi.1002830)
Supplement: Text S1 — Detailed description of ComiR implementation and other supporting information. (PDF) [file pcbi.1002830.s017.pdf]

Coronnello, Hartmaier, *et al.*, 2012,
"Novel modeling of combinatorial miRNA targeting identifies SNP with potential role in bone density"

SUPPLEMENTARY TEXT S1. Additional information on ComiR implementation.

Target prediction tools implementation

ComiR is based on the output score of four existing target prediction tools: PITA, miRanda, TargetScan and mirSVR. We obtained the output scores from PITA and TargetScan by running the public available software with the default parameters, with few exceptions. We ran miRanda with parameters "-en 0 –sc 0" so that it would return all the binding sites with energy score greater than 0. Predictions obtained with mirSVR were downloaded from the website http://www.microrna.org. We used the mirSVR prediction files labeled as "Good mirSVR score, Conserved miRNA". In ComiR, we used the following target scores, associated to each miRNA-3'UTR pair. (i) PITA: target scores = the difference between the free energy gained by the binding of the miRNA to the target and the free energy lost by unpairing the target-site nucleotides ($\Delta\Delta G$) of each binding site. (ii) miRanda: target score = the binding energy of each binding site. (iii) TargetScan: target score = number of detected binding sites. (no phylogenetic conservation features were used.) (iv) mirSVR: target score = mirSVR score of the predicted target sites.

Are the miRNA target score characteristics conserved across species?

The Drosophila Ago1-IP dataset is unique in that it contains abundance information for both Ago-bound mRNAs and miRNAs. So, the question becomes whether a Drosophila-trained model can perform well across species. To start investigating that, we examined the distribution of the scores of the four target prediction algorithms (PITA, miRanda, TargetScan, mirSVR) in three species: *D. melanogaster* (fly), *C. elegans* (worm), and *H.*

sapiens (human). In each species, we use all the available miRNA mature sequences, from miRBase.org, against all the available 3'UTR sequences, from Ensembl.org. We ran miRanda, PITA and TargetScan by matching all the possible pairs of miRNA and 3'UTR available in the three species. In Fig. S1 we report the density distributions of (A) the binding energy of all the binding sites as calculated with miRanda, (B) the $\Delta\Delta G$ as calculated by PITA for all the binding sites and (C) all the mirSVR scores of the predicted conserved target sites obtained with miRNAs, (D) the number of binding sites as detected by TargetScan. We found that the distribution of the target scores differs substantially between human and the two invertebrates. Human miRNA targets show lower binding energies (Fig. S1.A). Similarly, TargetScan plots show that human 3' UTRs tend to have more targets per 3'UTR than the worm or the fly (Fig. S1.D).

Normalization. Since the score distributions differ between humans and Drosophila, some normalization was required. We tested two kinds of normalization of the combined scores: (1) normalization by mean and (2) normalization by rank. The normalization by mean is performed by dividing the original combined scores by the mean of the combined score of all the considered genes, for each of the four considered tools. The normalization by rank is performed by considering the relative rank of the combined scores for each of the four considered tools. The rank normalization was slightly better (data not shown) and this is what we used in the rest of this manuscript.
